# Atypical antipsychotic medications and hyponatremia in older adults: a population-based cohort study

**DOI:** 10.1186/s40697-016-0111-z

**Published:** 2016-04-11

**Authors:** Sonja Gandhi, Eric McArthur, Jeffrey P. Reiss, Muhammad M. Mamdani, Daniel G. Hackam, Matthew A. Weir, Amit X. Garg

**Affiliations:** Department of Epidemiology and Biostatistics, Western University, London, Ontario Canada; Division of Nephrology, Department of Medicine, Western University, London, Ontario Canada; Institute for Clinical Evaluative Sciences, Toronto, Ontario Canada; Department of Psychiatry, Western University, London, Ontario Canada; Keenan Research Centre in the Li Ka Shing Knowledge Institute of St Michael’s Hospital, Toronto, Ontario Canada; Division of Clinical Pharmacology, Department of Medicine, Western University, London, Ontario Canada; Institute for Clinical Evaluative Sciences, Room ELL-101, Westminster, London Health Sciences Centre, 800 Commissioners Road East, London, Ontario N6A 4G5 Canada

**Keywords:** Atypical antipsychotic, Psychotropic, Risperidone, Olanzapine, Quetiapine, Hyponatremia, Low serum sodium

## Abstract

**Background:**

A number of case reports have suggested a possible association between atypical antipsychotic medications and hyponatremia. Currently, there are no reliable estimates of hyponatremia risk from atypical antipsychotic drugs.

**Objective:**

The objective of this study was to examine the 30-day risk of hospitalization with hyponatremia in older adults dispensed an atypical antipsychotic drug relative to no antipsychotic use.

**Design:**

The design of this study was a retrospective, population-based cohort study.

**Setting:**

The setting of this study was in Ontario, Canada, from 2003 to 2012.

**Patients:**

Adults 65 years or older with an identified psychiatric condition who were newly dispensed risperidone, olanzapine, or quetiapine in the community setting compared to adults with similar indicators of baseline health who were not dispensed such a prescription.

**Measurements:**

The primary outcome was the 30-day risk of hospitalization with hyponatremia. The tracer outcome (an outcome that is not expected to be influenced by the study drugs) was the 30-day risk of hospitalization with bowel obstruction. These outcomes were assessed using hospital diagnosis codes.

**Methods:**

Using health administrative data, we applied a propensity score technique to match antipsychotic users 1:1 to non-users of antipsychotic drugs (58,008 patients in each group). We used conditional logistic regression to compare outcomes among the matched users and non-users.

**Results:**

A total of 104 baseline characteristics were well-balanced between the two matched groups. Atypical antipsychotic use compared to non-use was associated with an increased risk of hospitalization with hyponatremia within 30 days (86/58,008 (0.15 %) versus 53/58,008 (0.09 %); relative risk 1.62 (95 % confidence interval (CI) 1.15 to 2.29); absolute risk increase 0.06 % (95 % CI 0.02 to 0.10)). The limited number of events precluded some additional analyses to confirm if the association was robust. Atypical antipsychotic use compared to non-use was not associated with hospitalization with bowel obstruction within 30 days (55/58,008 (0.09 %) versus 44/58,008 (0.08 %); relative risk 1.25 (95 % CI 0.84 to 1.86)).

**Limitations:**

We could only study older adults within our data sources.

**Conclusions:**

In this study, the use of an atypical antipsychotic was associated with a modest but statistically significant increase in the 30-day risk of a hospitalization with hyponatremia. The association was less pronounced than that described with other psychotropic drugs.

**Electronic supplementary material:**

The online version of this article (doi:10.1186/s40697-016-0111-z) contains supplementary material, which is available to authorized users.

## ABRÉGÉ

**Mise en contexte:**

De nombreux exposés de cas font état d’une possible corrélation entre la prise de médicaments antipsychotiques atypiques et l’hyponatrémie. Or actuellement, il n’existe aucune évaluation fiable qui vienne corroborer cette association.

**Objectifs de l’étude:**

L’étude visait à évaluer le risque, sur une période de 30 jours, d’hospitalisation pour cause d’hyponatrémie chez des patients adultes sous ordonnance d’un antipsychotique atypique, par rapport à un groupe de patients qui n’en consommaient pas.

**Cadre et type d’étude:**

Il s’agit d’une étude de cohorte, représentative de la population et rétrospective, qui s’est tenue en Ontario, au Canada, de 2003 à 2012.

**Participants:**

L’étude a porté sur un groupe d’adultes de plus de 65 ans avec un trouble psychiatrique établi et qui débutaient un traitement par risperidone, olanzapine ou quétiapine en milieu communautaire. Ils ont été comparés à un groupe d’individus ayant des indicateurs de santé initiaux équivalents, mais ne prenant pas d’antipsychotiques atypiques.

**Mesures:**

Le critère de jugement principal était le risque d’hospitalisation pour cause d’hyponatrémie à l’intérieur d’une période de 30 jours. À titre de traceur (événement sur lequel le médicament administré ne devait avoir aucune influence), on a utilisé le risque d’hospitalisation pour occlusion intestinale. Ces résultats ont été évalués en utilisant les codes de diagnostic des hôpitaux.

**Méthodologie:**

À l’aide des données administratives de la santé, la méthode statistique de l’appariement par scores de propension a été utilisée pour jumeler chaque patient sous médication antipsychotique avec un patient n’en consommant pas (58,008 patients dans chacun des groupes). On a eu recours à une régression logistique conditionnelle pour comparer les résultats observés au sein des couples de patients ainsi jumelés.

**Résultats:**

On a identifié 104 caractéristiques initiales bien balancées entre les deux groupes de participants. La prise d’antipsychotiques atypiques a été associée à un risque accru d’hospitalisation pour cause d’hyponatrémie à l’intérieur d’une période de 30 jours, lorsque comparée à la non-consommation (86/58 008 [0,15 %] contre 53/58 008 [0,09 %] ; risque relatif de 1,62 [95 % intervalle de confiance [IC] entre 1,15 et 2,29] ; augmentation absolue du risque à 0,06 % [95 % IC entre 0,02 et 0,10]. Le nombre limité d’événements empêche quelques analyses complémentaires qui permettraient de confirmer la robustesse de l’association entre l’hospitalisation pour hyponatrémie et la médication. Par ailleurs, la prise d’antipsychotiques atypiques n’a pas été associée à une hospitalisation pour obstruction intestinale pour la même période de 30 jours lorsque comparée au groupe de patients n’en consommant pas [55/58 008 [0,09 %] contre 44/58 008 [0,08 %] ; risque relatif de 1,25 [95 % IC entre 0,84 et 1,86]].

**Limites de l’étude:**

Les sources de données consultées n’ont permis que d’étudier les cas de patients adultes et âgés.

**Conclusions:**

Dans la présente étude, la consommation d’un médicament antipsychotique atypique a été associée à une légère, quoique significative, augmentation du risque d’être hospitalisé pour cause d’hyponatrémie, à l’intérieur d’une période de 30 jours. Toutefois, l’association s’est avérée moins marquée que pour d’autres médicaments psychotropes.

## What was known before

Hyponatremia following atypical antipsychotic use was observed in a number of case reports, but the risk is unknown.

### What this adds

This is a large population-based study to assess the risk of hyponatremia from atypical antipsychotics. The use of an atypical antipsychotic was associated with a modest but statistically significant increase in the 30-day risk of a hospitalization with hyponatremia compared to non-use.

## Introduction

Hyponatremia is one of the most frequently encountered electrolyte disorders in clinical practice and occurs in about 7 to 11 % of community-dwelling older adults [1]. Older adults are particularly susceptible to developing hyponatremia with age-related changes in homeostatic mechanisms and an increased number of comorbidities and concomitant medications known to cause hyponatremia [2]. As the sodium concentration falls below 135 mmol/L, there is a greater potential for clinical consequences such as confusion, seizures, respiratory arrest, fractures, or even death [3–6].

Atypical antipsychotic medications are routinely prescribed for the treatment of psychiatric disorders such as schizophrenia as well as the behavioral and psychological symptoms of dementia. The use of atypical antipsychotics has been rising, with nearly 15 million prescriptions dispensed in Canada in 2012 [7, 8]. Frequently prescribed atypical antipsychotics include risperidone (risperdal®), olanzapine (zyprexa®), and quetiapine (seroquel®). A number of case reports have suggested a possible association between atypical antipsychotics and hyponatremia [9–14]. Hyponatremia from older typical antipsychotics has also been observed, but their use has been declining in routine care, primarily due to their adverse side effect profile [7, 15, 16]. Like other psychotropic medications, it is suspected that atypical antipsychotics can induce hyponatremia by either stimulating antidiuretic hormone release from the brain or enhancing antidiuretic hormone activity in the kidneys [13].

Currently, there are no reliable estimates of incidence or risk of hyponatremia from atypical antipsychotic drugs (risperidone, olanzapine, and quetiapine) in older adults. To date, only one case-control study has examined the association between atypical antipsychotic drug use and hyponatremia [17]. This study looked at a series of voluntary reports of adverse drug reactions made to the World Health Organization and found that the use of olanzapine and risperidone (as well as other atypical antipsychotics) was associated with more reporting of hyponatremia compared to other adverse drug reactions. However, data from spontaneous reporting systems is subject to biases [18]. This may be one reason why the risk of hyponatremia from atypical antipsychotics is reported inconsistently across popular drug prescribing references, such as UpToDate® and the Canadian Compendium of Pharmaceuticals and Specialties. We conducted this population-based cohort study in older adults to understand the association between the new use of atypical antipsychotics and the 30-day risk of hospitalization with hyponatremia relative to non-users of atypical antipsychotics.

## Methods

### Study design and setting

We used multiple linked health administrative databases to conduct a retrospective population-based cohort study of older adults from June 2003 to March 2012 in Ontario, Canada. There are over two million residents 65 years and older in Ontario who have universal access to hospital care, physician services, and prescription drug coverage [19]. These datasets were linked using unique encoded identifiers and were analyzed at the Institute for Clinical Evaluative Sciences (ICES). We conducted this study according to a pre-specified protocol that was approved by the institutional review board at Sunnybrook Health Sciences Centre, Toronto, Canada. The reporting of this study followed the Strengthening the Reporting of Observational Studies in Epidemiology guidelines (Additional file 1: Table S1) [20].

### Data sources

We identified information related to patients, medications, covariates, and outcomes from nine linked databases. For all residents with a valid provincial health card, we obtained demographic information and vital statistics using the Ontario Registered Persons Database. We used the Ontario Drug Benefits Program database to identify exposure to atypical antipsychotics and other medications. This database accurately records prescription claims for outpatients over the age of 65 (error rate of 0.7 %) [21]. We defined covariates using the Ontario Health Insurance Plan database, which contains information on health claims for inpatient and outpatient physician services. We identified diagnostic and procedural information on all hospitalizations and emergency department visits using the Canadian Institute for Health Information’s Discharge Abstract Database and National Ambulatory Care Reporting System database, respectively. Similarly, we used the Ontario Mental Health Reporting System database to obtain mental health information. We obtained antipsychotic prescriber information from the ICES Physician Database. We identified serum sodium measurements using datasets from Gamma-Dynacare Medical Laboratories (a laboratory service provider in Ontario) and Cerner (an electronic medical record system that is used by 12 hospitals in southwestern Ontario). We have used these databases to research adverse drug events and health outcomes in several other studies (including outcomes of hyponatremia and health services) [22–26].

The databases were complete for all variables considered in this study, with the exception of income quintile, rural residence, and prescriber information. We used codes from the *International Classification of Diseases, 9th revision (ICD-9)* and *10th revision (ICD-10)* to ascertain baseline comorbidities in the 5 years prior to cohort entry. Outcomes were identified using only *ICD-10* codes as these events would have occurred following implementation of this coding system in 2002. The diagnostic codes used in our study are detailed in Additional file 1: Table S2.

### Cohort

For our exposed group, we considered all older adults in Ontario who had evidence of a hospital diagnosis or physician claim for a psychiatric condition (dementia, schizophrenia, bipolar disorder, unipolar depression/anxiety, or Parkinson’s disease) within the previous 5 years and who commenced treatment with risperidone, olanzapine, or quetiapine (users). These are the atypical antipsychotics used most frequently in our region. We defined new use as no prescriptions for any type of antipsychotic drug in the prior 6 months. Patients could only be prescribed one atypical antipsychotic so that we could compare mutually exclusive groups in subgroup analyses. The date of the prescription served as the index date (cohort entry date). We then identified a referent group of older adults with a psychiatric condition, as defined above, from the Ontario population who were not prescribed any kind of antipsychotic (non-users). We randomly assigned an index date to non-users based on the distribution of index dates for the users.

We excluded the following individuals from analyses: (1) patients discharged from hospital in the 2 days prior to their index date to ensure new outpatient antipsychotic use (in the case of the users; or the possibility of a new outpatient antipsychotic prescription in the case of non-users) and (2) patients with end-stage renal disease prior to their index date since serum sodium levels are controlled through dialysis. We excluded all patients with no outpatient medications of any kind dispensed in the 90 days prior to their index date to ensure all were active users of the Ontario Drug Benefits program.

Using a logistic regression model, we derived a propensity score for the predicted probability of commencing treatment with an atypical antipsychotic drug. The propensity score included more than 100 variables that were potentially associated with atypical antipsychotic drug use or hospitalization with hyponatremia (Additional file 1: Table S3) [27]. Then, using greedy matching, we matched 1:1 each atypical antipsychotic drug user to a non-user based on the following characteristics: age (within 2 years); sex; index date (within 1 year); residential status (community-dwelling or long-term care); dementia, schizophrenia, bipolar disorder, unipolar depression/anxiety, Parkinson’s disease, chronic kidney disease, and congestive heart failure; diuretic use; constituency in the catchment area where linked serum sodium data were available; and the logit of the propensity score (within a caliper of ±0.2 standard deviations). A patient could only enter the study once.

### Primary outcome

We evaluated the primary outcome of hospitalization with hyponatremia within 30 days following the index date. Hospitalization with any evidence of *ICD-10* code E87.1 (hypo-osmolality or hyponatremia) in the patient’s record was included (any one of 25 diagnoses). Based on a validation study conducted in our region, the presence of code E87.1 in a given hospitalization identifies older patients with a median serum sodium value of 123 mmol/L at hospital admission (interquartile range (IQR) 119 to 126 mmol/L), whereas its absence identifies a median value of 138 mmol/L (IQR 136 to 140 mmol/L). The specificity of the code is over 99 %, while sensitivity is 11 % for hyponatremia defined as a serum sodium concentration ≤ 132 mmol/L. The sensitivity of the code increases when hyponatremia is defined by lower serum sodium concentrations [28].

### Statistical analyses

We used standardized differences to compare baseline characteristics between user and non-user groups. This metric describes differences between the group means relative to the pooled standard deviation with a value less than 10 % indicating adequate balance [29]. We used conditional logistic regression to estimate odds ratios and 95 % confidence intervals (CIs). We evaluated the association between atypical antipsychotic drug use and a hospitalization with hyponatremia in the following pre-specified subgroups: (1) antipsychotic medication type (risperidone, quetiapine, or olanzapine), (2) antipsychotic dose (higher dose versus normal dose; higher dose defined by a higher than median starting daily dose for the study cohort) (Additional file 1: Table S2), (3) chronic kidney disease, (4) congestive heart failure, and (5) diuretic use. We identified chronic kidney disease and congestive heart failure using separate validated algorithms of hospital diagnostic codes [30, 31]. In the case of antipsychotic type and dose, we defined the subgroup by the characteristic in users with non-users following their matched user. We determined subgroup *p* values using interaction terms in the logistic regression models.

### Additional analyses

We tested the specificity of our findings by evaluating the 30-day risk of a hospitalization with bowel obstruction in the two groups. We expected antipsychotic use would not alter the risk of bowel obstruction and reasoned that a null association with this outcome would enhance causal inference in our hyponatremia analyses. We re-evaluated the outcome of hospitalization with hyponatremia in our existing cohort at a time that preceded the index date by 90 days (a time when no patient would have been prescribed an antipsychotic). After re-applying exclusion criteria at this time, we followed retained matched pairs to re-assess the 30-day risk. In this analysis, a null association would enhance the assertion that the two groups were similar in their baseline risk for hyponatremia in the absence of antipsychotic medication use. We also examined the risk factors associated with hospitalization with hyponatremia separately in users and non-users using multiple logistic regression. The risk factors that we considered were age (per year); sex; chronic kidney disease, congestive heart failure, diabetes, liver disease, cancer, hypothyroidism, and previous hyponatremia; and receipt of a diuretic, antiepileptic, antidepressant, and antineoplastic drugs. We expressed risk in both relative and absolute terms. All odds ratios were approximated as relative risks (appropriate given the incidences observed). We performed all analyses using SAS version 9.4 (SAS Institute, Cary, North Carolina).

## Results

Prior to matching, we identified 92,090 antipsychotic users and 175,836 non-users who were eligible for our study. Cohort selection and baseline characteristics are presented in Additional file 1: Figure S1 and Table 1, respectively. In the unmatched cohort, antipsychotic users compared to non-users were more likely to be older and reside in a long-term care facility and prior to cohort entry were more likely to receive a greater number of medications and health-care services compared to non-users. We successfully matched 58,008 users to 58,008 non-users, and baseline characteristics were well-balanced between the two groups (104 characteristics measured; full baseline table is presented in Additional file 1: Table S4). The mean age was 81 years, and 67 % were women. Nearly 48 % of the users were prescribed risperidone, and family physicians wrote 70 % of the prescriptions.

**Table 1:** Baseline characteristics of atypical antipsychotic users and non-users

Characteristic	Unmatched			Matched		
	Antipsychotic users	Antipsychotic non-users	Standardized difference^a^	Antipsychotic users	Antipsychotic non-users	Standardized difference^a^
	(*n* = 92,090)	(*n* = 175,836)		(*n* = 58,008)	(*n* = 58,008)	
Demographic						
Age, mean (SD), years	81 (7.8)	79 (7.9)	29.2 %	81 (7.7)	81 (7.7)	0.3 %
Women	58,647 (63.7 %)	111,968 (63.7 %)	0 %	38,736 (66.8 %)	38,736 (66.8 %)	0 %
Income quintile^b^						
1 (low)	20,160 (21.9 %)	37,436 (21.3 %)	1.5 %	12,331 (21.3 %)	13,081 (22.6 %)	3.1 %
2	18,854 (20.5 %)	36,395 (20.7 %)	0.6 %	11,888 (20.5 %)	12,057 (20.8 %)	0.7 %
3 (medium)	17,999 (19.6 %)	33,861 (19.3 %)	0.7 %	11,630 (20.1 %)	11,408 (19.7 %)	1.0 %
4	17,607 (19.1 %)	33,206 (18.9 %)	0.6 %	11,213 (19.3 %)	10,847 (18.7 %)	1.6 %
5 (high)	17,058 (18.5 %)	34,373 (19.6 %)	2.6 %	10,946 (18.9 %)	10,615 (18.3 %)	1.5 %
Rural residence	11,759 (12.8 %)	23,484 (13.4 %)	1.7 %	7671 (13.2 %)	7557 (13.0 %)	0.6 %
Long-term care	32,644 (35.5 %)	26,705 (15.2 %)	47.9 %	16,409 (28.3 %)	16,409 (28.3 %)	0 %
Comorbid conditions^c^						
Charlson comorbidity index, mean (SD)	1.68 (1.8)	1.56 (1.8)	6.7 %	0.87 (1.5)	0.94 (1.5)	4.7 %
Johns Hopkins ACG System Aggregated Diagnosis Groups, mean (SD)	13.90 (4.2)	13.69 (4.0)	5.2 %	13.37 (4.2)	13.69 (4.1)	7.8 %
Dementia	71,933 (78.1 %)	92,049 (52.4 %)	56.2 %	44,715 (77.1 %)	44,715 (77.1 %)	0 %
Schizophrenia	14,838 (16.1 %)	14,072 (8.0 %)	25.1 %	4756 (8.2 %)	4756 (8.2 %)	0 %
Bipolar disorder	10,174 (11.1 %)	11,377 (6.5 %)	16.3 %	3295 (5.7 %)	3295 (5.7 %)	0 %
Unipolar depression/anxiety	28,419 (30.9 %)	74,574 (42.4 %)	24.2 %	15,038 (25.9 %)	15,038 (25.9 %)	0 %
Parkinson’s disease	8652 (9.4 %)	19,015 (10.8 %)	4.7 %	3780 (6.5 %)	3780 (6.5 %)	0 %
Congestive heart failure	19,029 (20.7 %)	33,627 (19.1 %)	3.9 %	10,038 (17.3 %)	10,038 (17.3 %)	0 %
Chronic kidney disease	8127 (8.8 %)	15,323 (8.7 %)	0.4 %	3140 (5.4 %)	3140 (5.4 %)	0 %
Hypertension	65,205 (70.8 %)	131,562 (74.8 %)	9.0 %	40,929 (70.6 %)	40,419 (69.7 %)	1.9 %
Chronic liver disease	2980 (3.2 %)	6388 (3.6 %)	2.2 %	1664 (2.9 %)	1807 (3.1 %)	1.5 %
Hypothyroidism	10,213 (11.1 %)	20,354 (11.6 %)	1.5 %	6222 (10.7 %)	6198 (10.7 %)	0.1 %
Cancer	12,145 (13.2 %)	25,758 (14.7 %)	4.2 %	7321 (12.6 %)	7864 (13.6 %)	2.8 %
Diabetes mellitus	14,245 (15.5 %)	30,491 (17.3 %)	5.1 %	17,590 (30.3 %)	18,457 (31.8 %)	3.2 %
Pneumonia	8006 (8.7 %)	12,843 (7.3 %)	5.1 %	4237 (7.3 %)	4755 (8.2 %)	3.3 %
Coronary artery disease^d^	31,417 (34.1 %)	61,334 (34.9 %)	1.6 %	18,641 (32.1 %)	19,184 (33.1 %)	2.0 %
Angina	20,496 (22.3 %)	42,264 (24.0 %)	4.2 %	12,166 (21.0 %)	12,462 (21.5 %)	1.3 %
Previous hyponatremia	3403 (3.7 %)	5416 (3.1 %)	3.4 %	1766 (3.0 %)	2111 (3.6 %)	3.3 %
Lung disease	26,237 (28.5 %)	53,842 (30.6 %)	4.7 %	15,489 (26.7 %)	16,891 (29.1 %)	5.4 %
Seizure	1782 (1.9 %)	2780 (1.6 %)	2.7 %	879 (1.5 %)	1087 (1.9 %)	2.8 %
Acute kidney injury	3453 (3.8 %)	5616 (3.2 %)	3.0 %	1482 (2.6 %)	1501 (2.6 %)	0.2 %
Acute urinary retention	3337 (3.6 %)	5179 (3.0 %)	3.8 %	1650 (2.8 %)	1774 (3.1 %)	1.3 %
Delirium	7112 (7.7 %)	6013 (3.4 %)	18.8 %	3424 (5.9 %)	2546 (4.4 %)	6.9 %
Peripheral vascular disease	1939 (2.1 %)	4061 (2.3 %)	1.4 %	1043 (1.8 %)	1238 (2.1 %)	2.4 %
Concurrent medication use^e^						
Number of unique drug products, mean (SD)	9.71 (6.4)	8.94 (5.5)	13.0 %	8.91 (5.9)	9.41 (5.6)	8.7 %
Anticonvulsants	10,970 (11.9 %)	16,409 (9.3 %)	8.4 %	5552 (9.6 %)	6688 (11.5 %)	6.4 %
Antidepressants	46,600 (50.6 %)	65,227 (37.1 %)	27.5 %	25,197 (43.4 %)	26,871 (46.3 %)	5.8 %
Antidiabetics	14,245 (15.5 %)	21,969 (12.5 %)	8.6 %	8526 (14.7 %)	9307 (16.0 %)	3.7 %
Antineoplastics	3240 (3.5 %)	6863 (3.9 %)	2.0 %	1958 (3.4 %)	2151 (3.7 %)	1.8 %
Thyroxine	16,580 (18.0 %)	33,499 (19.1 %)	2.7 %	10,406 (17.9 %)	10,846 (18.7 %)	2.0 %
Potassium sparing diuretics	5390 (5.9 %)	11,025 (6.3 %)	1.8 %	3267 (5.6 %)	3236 (5.6 %)	0.2 %
Non-potassium sparing diuretics	31,051 (33.7 %)	61,131 (34.8 %)	2.2 %	18,611 (32.1 %)	18,665 (32.2 %)	0.2 %
ACE inhibitors and/or ARBs	39,596 (43.0 %)	88,563 (50.4 %)	14.8 %	24,853 (42.8 %)	25,660 (44.2 %)	2.8 %
NSAIDs (excl. ASA)	13,638 (14.8 %)	30,146 (17.1 %)	6.4 %	8667 (14.9 %)	9047 (15.6 %)	1.8 %
Calcium channel blockers	22,902 (24.9 %)	49,786 (28.3 %)	7.8 %	14,642 (25.2 %)	15,011 (25.9 %)	1.5 %
Beta-adrenergic agonists	26,099 (28.3 %)	55,259 (31.4 %)	6.7 %	16,169 (27.9 %)	16,254 (28.0 %)	0.3 %
Statins	29,558 (32.1 %)	72,878 (41.5 %)	19.5 %	19,171 (33.1 %)	19,514 (33.6 %)	1.3 %
Benzodiazepines	33,031 (35.9 %)	45,988 (26.2 %)	21.1 %	17,616 (30.4 %)	18,692 (32.2 %)	4.0 %
Healthcare contacts, mean (SD)^f^						
Hospitalizations	0.51 (0.9)	0.32 (0.8)	22.6 %	0.40 (0.8)	0.39 (0.8)	1.3 %
Emergency department visits	1.29 (2.1)	0.84 (1.6)	24.7 %	1.04 (1.6)	1.00 (1.7)	2.5 %
Family physician visits	18.57 (18.0)	13.61 (13.1)	31.9 %	15.84 (15.5)	16.30 (15.3)	3.0 %
Geriatrician visits	0.82 (3.6)	0.34 (2.1)	16.9 %	0.58 (2.5)	0.49 (2.4)	3.7 %
Psychiatrist visits	1.69 (7.5)	0.36 (2.5)	26.6 %	0.64 (2.4)	0.40 (2.2)	10.0 %
Health-care use^g^						
Previous sodium tests	63,335 (68.8 %)	79,930 (45.5 %)	48.5 %	38,190 (64.8 %)	38,145 (65.8 %)	0.2 %
Carotid ultrasound	4568 (5.0 %)	9137 (5.2 %)	1.1 %	2721 (4.7 %)	2883 (5.0 %)	1.3 %
Cardiac catheterization	742 (0.8 %)	2334 (1.3 %)	5.1 %	414 (0.7 %)	477 (0.8 %)	1.2 %
Echocardiography	12,411 (13.5 %)	27,264 (15.5 %)	5.8 %	7247 (12.5 %)	7355 (12.7 %)	0.6 %
Holter monitoring	4818 (5.2 %)	10,904 (6.2 %)	4.2 %	2962 (5.1 %)	3032 (5.2 %)	0.6 %
Colorectal cancer screening	10,653 (11.6 %)	29,013 (16.5 %)	14.2 %	6767 (11.7 %)	6823 (11.8 %)	0.3 %
Cervical cancer screening	2047 (2.2 %)	8290 (4.7 %)	13.7 %	1447 (2.5 %)	1427 (2.5 %)	0.2 %
Thyroid stimulating hormone	57,414 (62.4 %)	100,900 (57.4 %)	10.1 %	34,911 (60.2 %)	34,766 (59.9 %)	0.5 %
Bone mineral density test	5792 (6.3 %)	18,558 (10.6 %)	15.4 %	4040 (7.0 %)	4110 (7.1 %)	0.5 %
Hearing test	3705 (4.0 %)	9327 (5.3 %)	6.1 %	2375 (4.1 %)	2537 (4.4 %)	1.4 %
Cytoscopy	3959 (4.3 %)	7739 (4.4 %)	0.5 %	2253 (3.9 %)	2325 (4.0 %)	0.6 %
Computed tomography of the head	26,927 (29.2 %)	25,724 (14.6 %)	35.9 %	13,261 (22.9 %)	12,896 (22.2 %)	1.5 %
Chest X-ray	43,501 (47.2 %)	69,077 (39.3 %)	16.1 %	24,600 (42.4 %)	25,086 (43.3 %)	1.7 %
Pulmonary function test	4882 (5.3 %)	13,764 (7.8 %)	10.2 %	2901 (5.0 %)	3404 (5.9 %)	3.8 %
Laboratory measurements^h^						
Evidence of baseline serum sodium measurement, *N* (%)	14,346 (15.6 %)	21,948 (23.8 %)	20.9 %	7242 (12.5 %)	7242 (12.5 %)	0 %
Most recent serum sodium, mean (SD)	140.3 (3.5)	140.4 (3.2)	2.7 %	140.4 (3.4)	140.3 (3.4)	4.4 %

Data are presented as the number (percentage) of patients, unless otherwise reported

*ACE inhibitor* angiotensin-converting enzyme inhibitor, *ACG* adjusted clinical groups, *ARB* angiotensin II receptor blocker, *IQR* interquartile range, *NSAID* non-steroidal anti-inflammatory drug, *SD* standard deviation

^a^Standardized differences are less sensitive to sample size than traditional hypothesis tests. They provide a measure of the difference between groups with respect to the pooled standard deviation; a standardized difference greater than 10 % was considered as a meaningful difference between the groups

^b^Income was categorized into fifths of average neighborhood income on the index date

^c^Comorbid conditions in the 5 years preceding the index date were considered

^d^Coronary artery disease includes receipt of coronary artery bypass graft surgery and percutaneous coronary intervention

^e^Concurrent medication use in the 6 months preceding the index date were considered

^f^Health-care contacts in the year preceding the index date were considered

^g^Health-care use in the year preceding the index date was considered

^h^Serum sodium measurements were obtained at a mean (SD) of 140 (102) days in users and 149 (101) days in non-users, prior to the index date

### Primary outcome

Results for the primary outcome are presented in Table 2. Atypical antipsychotic use was associated with a greater 30-day risk of hospitalization with hyponatremia compared to non-use (86/58,008 (0.15 %) versus 53/58,008 (0.09 %); relative risk 1.62 (95 % CI 1.15 to 2.29), absolute risk increase 0.06 % (95 CI 0.02 to 0.10 %)).

**Table 2:** 30-day risk of a hospitalization with hyponatremia (as defined by a hospital diagnosis code) in antipsychotic drug users and non-users

	Events, no. (%)^a^		Relative risk (95 % CI)	Absolute risk increase (95 % CI), %
	Antipsychotic users	Antipsychotic non-users^b^		
	(*n* = 58,008)	(*n* = 58,008)		
Hospitalization with hyponatremia^c^	86 (0.15)	53 (0.09)	1.62 (1.15 to 2.29)	0.06 (0.02 to 0.10)

*CI* confidence interval

^a^The event rates and absolute risk differences are underestimated as the hospital-based diagnosis codes used to define the outcomes have high specificity but low sensitivity

^b^An antipsychotic non-users group was the referent

^c^The sensitivity and specificity of the code for hyponatremia is 11 and 99 %, respectively [30]

**Fig. 1:**
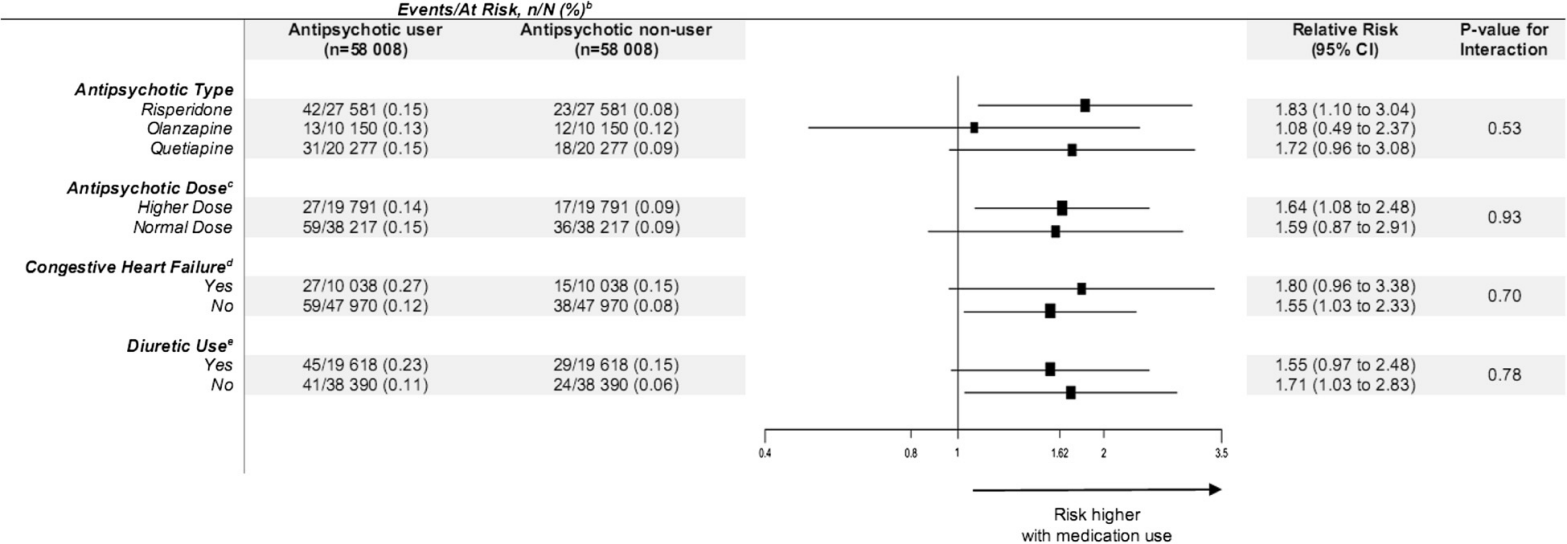
The association between antipsychotic use and hospitalization with hyponatremia assessed in four subgroups^a^. *CI* confidence interval. *a* Antipsychotic type, antipsychotic dose, chronic kidney disease, congestive heart failure, and use of a diuretic. Sets of medication users and non-users were matched on presence of chronic kidney disease, congestive heart failure, and baseline diuretic use. For antipsychotic type and dose, matched sets were categorized according to this characteristic in users. *b* Hyponatremia (and the proportion of patients who had an event) was assessed by using a hospital diagnosis code. The true event rate of hyponatremia is underestimated for some outcomes because the code for hyponatremia has high specificity but low sensitivity. *c* Higher dose was defined as a higher than median daily dose. See Additional file 1: Table S2 for definitions. *d* Congestive heart failure has a sensitivity, specificity, and positive predictive value of 84.3, 85.4, and 35.8 %, respectively [31]. *e* Diuretic use includes potassium sparing and non-potassium sparing medications. Chronic kidney disease was included in the test for interactions but was removed from presentation as there were too few events for meaningful analysis. This was also done to comply with privacy regulations, to prevent the risk of re-identification when the size of the numerator is small (less than or equal to 5). Chronic kidney disease was identified by using an algorithm of hospital diagnosis codes validated for older adults in the study region [30]. The algorithm identified patients with a median estimated glomerular filtration rate of 38 mL/min/1.73 m^2^ (interquartile range, 27–52 mL/min/1.73 m^2^), whereas its absence identified patients with a median estimated glomerular filtration rate of 69 mL/min/1.73 m^2^ (interquartile range, 56–82 mL/min/1.73 m^2^). Data marker size is proportional to the inverse of the source variance

Results from subgroup analyses are presented in Fig. 1. There were too few events among patients with chronic kidney disease for meaningful subgroup analysis, and to minimize any risk of patient re-identification in our data sources, we were not permitted to report results when a group of events were less than or equal to 5. The association between atypical antipsychotic drug use and hospitalization with hyponatremia was not influenced by the atypical antipsychotic drug, dose, congestive heart failure, or diuretic use (all *p* values for interaction > 0.05).

### Additional analyses

The risk of a hospitalization with bowel obstruction was not significantly different between atypical antipsychotic users and non-users (55/58,008 (0.09 %) versus 44/58,008 (0.08 %); relative risk 1.25 (95 % CI 0.84 to 1.86); absolute risk increase 0.02 % (95 % CI −0.01 to 0.05 %)).

Baseline characteristics were very similar in the cohort that was assessed in the 90 days prior to the index date (Additional file 1: Table S5; 42,698 retained matched pairs of users and non-users). When we re-examined the 30-day risk of a hospitalization with hyponatremia at that time, we did not observe a significant difference between users and non-users (relative risk 1.19 (95 % CI 0.74 to 1.92)) (Table 3).

**Table 3:** 30-day risk of a hospitalization with bowel obstruction (as defined by a hospital diagnosis code) in antipsychotic drug users and non-users

	Events, no. (%)^a^		Relative risk (95 % CI)	Absolute risk increase (95 % CI), %
	Antipsychotic users	Antipsychotic non-users^b^		
	(*n* = 58,008)	(*n* = 58,008)		
Hospitalization with bowel obstruction	55 (0.09)	44 (0.08)	1.25 (0.84 to 1.86)	0.02 (−0.01 to 0.05)

*CI* confidence interval

^a^The event rates and absolute risk differences are underestimated as the hospital-based diagnosis codes used to define the outcomes have high specificity but low sensitivity

^b^An antipsychotic non-users group was the referent

In our cohort, older age, cancer, and prior hyponatremia were significant risk factors for future hyponatremia in antipsychotic users (Additional file 1: Table S6).

## Discussion

Among older adults prescribed an atypical antipsychotic drug, we observed a modest increase in the relative and absolute risks of hospitalization with hyponatremia in users compared to non-users.

The results of this population-based study inform us about the nature of the association between atypical antipsychotic medications and hyponatremia. Our estimate is similar to that obtained in a previous case-control study that used individual case safety reports of hyponatremia to estimate a “reporting odds ratio” of 1.55 (95 % CI 1.41 to 1.69) [17]. This is a measure of disproportionality that estimates the extent to which hyponatremia is reported in association with an atypical antipsychotic medication relative to reports of hyponatremia with other medications. The low absolute risk observed in our study is likely influenced by the low sensitivity of the hospital diagnosis code for hyponatremia (~11 %), which underestimates the true incidence by up to eightfold [28]. Currently, UpToDate®, a popular reference widely used by physicians, warns of the possibility of hyponatremia and recommends monitoring the concentration of serum sodium in older adults upon initiation of an atypical antipsychotic medication [32–34]. Another important physician reference in our region, the Canadian Compendium of Pharmaceuticals and Specialties, does not provide any information or recommendations related to hyponatremia with atypical antipsychotic medications [35–37]. These product monographs should be updated to include the risk of hyponatremia.

Unlike with other psychotropic medications, such as selective serotonin or norepinephrine reuptake inhibitors (second-generation antidepressants), there is only a modest risk of hospitalization with hyponatremia from atypical antipsychotic drugs. For example, we recently conducted a study using similar methodology to assess the 30-day risk of hospitalization with hyponatremia following antidepressant drug use (unpublished data). In that study, the risk of hospitalization with hyponatremia was much higher in antidepressant users compared to non-users (relative risk 5.46 (95 % CI 4.32 to 6.91)). Consistent results were obtained when we assessed the 30-day risk of a hospitalization with hyponatremia using inpatient serum sodium measurements in a subpopulation that had laboratory data available (small catchment area of 12 hospitals comprising approximately 5 % of the total cohort) [38]. In the current study, we could not corroborate our primary findings using laboratory data as there were too few events in this subpopulation. Additionally, we could not confirm if patients who did not present to hospital had hyponatremia, as again, there were a limited number of events to evaluate the outcome of outpatient hyponatremia (using data from outpatient laboratories). It would also have been useful to know if the hyponatremia observed in our study was symptomatic (i.e., if patients presented to hospital with both hyponatremia and delirium). However, this too was not possible given the limited number of events.

There are important strengths of our study. The use of Ontario’s health-care databases including data on universal prescription drug coverage allowed us to estimate a rare adverse event with good precision in a large representative sample size. We used a propensity score-matched design to reduce confounding that is often found in observational studies. In addition, the results of our two additional analyses support our primary study finding. The null association seen with atypical antipsychotic use and bowel obstruction provides some reassurance that residual confounding is unlikely to have influenced the primary results.

There are some limitations of our study. First, as the sensitivity of the hospital diagnosis code for hyponatremia was low, we would have preferred to supplement our primary findings using serum sodium laboratory values. With codes however, we were able to capture those patients whose hyponatremia would be considered clinically significant (median serum sodium level of 123 mmol/L (IQR 119–126 mmol/L) as found in our validation study). Second, although our user and non-user groups were well-balanced after matching, unmeasured confounding variables may have influenced our estimates of risk. It is possible that the patients who used atypical antipsychotics in our study were more likely than non-users to experience psychogenic polydipsia, a condition characterized by excessive fluid consumption [39]. Third, we could not be confident of the indication for which the atypical antipsychotic drug was prescribed. Both users and non-users had evidence of a psychiatric condition in the previous 5 years. This restriction was applied to ensure that we had a large representative sample of similar types of patients in our study. Fourth, we were unable to look at the long-term risk of hyponatremia, because the median length of time in follow-up for antipsychotic use was only 57 days. When an antipsychotic is prescribed, only short duration use is encouraged, particularly in those with dementia (6–12 weeks), which is an unapproved indication [40, 41]. Alternatively, poor adherence to the medication could also explain the short duration observed [42, 43]. Finally, we could only study older adults within our data sources. Younger patients are often healthier and may be less susceptible to drug-induced hyponatremia.

To our knowledge, this is the first population-based study to examine the association between atypical antipsychotic medication use and hyponatremia among older adults. We recommend that additional studies be conducted in this area. Future studies should consider using serum sodium laboratory values to better estimate the risk of hyponatremia from atypical antipsychotics. In patients who are chronic users of antipsychotics, future studies should examine whether there is a long-term risk of hyponatremia. Even mild, chronic forms of hyponatremia can have important consequences, negatively impacting quality of life [44]. Better knowledge of these risks can help to guide medication prescribing and monitoring and interventions to prevent or mitigate adverse drug events. For now, we recommend judicious prescribing of atypical antipsychotic drugs to minimize adverse events. When a patient presents with severe hyponatremia, antipsychotic drugs can be considered as a potential reason for the finding.

## Conclusions

We found a modest increase in the 30-day risk of hospitalization with hyponatremia among older adults prescribed atypical antipsychotic medications compared to those who were not. The association was less pronounced than seen with other psychotropic drugs. Additional studies are needed to ensure reproducibility of the findings.

## Additional file

Additional file 1: Table S1–S6 and Figure S1. Table S1. STROBE Checklist. Table S2. Coding definitions for comorbid conditions, outcomes and exposures. Table S3. Variables included in propensity score model. Figure S1. Cohort selection. Table S4. Full baseline characteristics of atypical antipsychotic medication users and non-users. Table S5. Baseline characteristics of matched atypical antipsychotic users and non-users at 90 days prior to the index date. Table S6. Risk factors for hospitalization with hyponatremia in antipsychotic medication users and non-users. (DOCX 87 kb)
