# Supplementary material for: Atypical antipsychotic medications and hyponatremia in older adults: a population-based cohort study
Source: Can J Kidney Health Dis. 2016 Apr 11;3:21. doi: 10.1186/s40697-016-0111-z (PMC4827184; doi:10.1186/s40697-016-0111-z)
Supplement: Additional file 1: Table S1–S6 and Figure S1. — Table S1. STROBE Checklist. Table S2. Coding definitions for comorbid conditions, outcomes and exposures. Table S3. Variables included in propensity score model. Figure S1. Cohort selection. Table S4. Full baseline characteristics of atypical antipsychotic medication users and non-users. Table S5. Baseline characteristics of matched atypical antipsychotic users and non-users at 90 days prior to the index date. Table S6. Risk factors for hospitalization with hyponatremia in antipsychotic medication users and non-users. (DOCX 87 kb) [file 40697_2016_111_MOESM1_ESM.docx]

**Additional file 1**: **Table S1. STROBE Checklist**

|  | Item No | Recommendation | Reported |
| --- | --- | --- | --- |
| Title and abstract | 1 | (a) Indicate the study’s design with a commonly used term in the title or the abstract | Title, Abstract |
|  |  | (b) Provide in the abstract an informative and balanced summary of what was done and what was found | Abstract |
| Introduction | | | |
| Background/rationale | 2 | Explain the scientific background and rationale for the investigation being reported | Introduction |
| Objectives | 3 | State specific objectives, including any pre-specified hypotheses | Introduction |
| Methods | | | |
| Study design | 4 | Present key elements of study design early in the paper | Methods |
| Setting | 5 | Describe the setting, locations, and relevant dates, including periods of recruitment, exposure, follow-up, and data collection | Methods |
| Participants | 6 | (a) Give the eligibility criteria, and the sources and methods of selection of participants. Describe methods of follow-up | Methods, Appendix Figure 1 |
|  |  | (b) For matched studies, give matching criteria and number of exposed and unexposed | Methods, Table 1, Appendix Table 3, 4, 5 |
| Variables | 7 | Clearly define all outcomes, exposures, predictors, potential confounders, and effect modifiers. Give diagnostic criteria, if applicable | Methods, Appendix Table 2 |
| Data sources/measurement | 8 | For each variable of interest, give sources of data and details of methods of assessment (measurement). Describe comparability of assessment methods if there is more than one group | Methods, Appendix Tables 2 |
| Bias | 9 | Describe any efforts to address potential sources of bias | Discussion |
| Study size | 10 | Explain how the study size was arrived at | Methods; based on availability of the data |
| Quantitative variables | 11 | Explain how quantitative variables were handled in the analyses. If applicable, describe which groupings were chosen and why | Methods |
| Statistical methods | 12 | (a) Describe all statistical methods, including those used to control for confounding | Methods |
|  |  | (b) Describe any methods used to examine subgroups and interactions | Methods |
|  |  | (c) Explain how missing data were addressed | Methods |
|  |  | (d) If applicable, explain how loss to follow-up was addressed | Not applicable |
|  |  | (e) Describe any sensitivity analyses | Methods |
| Results | | | |
| Participants | 13 | (a) Report numbers of individuals at each stage of study—e.g. numbers potentially eligible, examined for eligibility, confirmed eligible, included in the study, completing follow-up, and analyzed | Methods, Results, Appendix Figure 1 |
|  |  | (b) Give reasons for non-participation at each stage | Methods, Appendix Figure 1 |
|  |  | (c) Consider use of a flow diagram | Appendix Figure 1 |
| Descriptive data | 14 | (a) Give characteristics of study participants (e.g. demographic, clinical, social) and information on exposures and potential confounders | Table 1, Appendix Tables 4, 5 |
|  |  | (b) Indicate number of participants with missing data for each variable of interest | Not applicable |
|  |  | (c) Summarize follow-up time (e.g. average and total amount) | Results, Table 2 |
| Outcome data | 15 | Report numbers of outcome events or summary measures over time | Results, Table 2, Figure 1 |
| Main results | 16 | (a) Give unadjusted estimates and, if applicable, confounder-adjusted estimates and their precision (e.g. 95% confidence interval). Make clear which confounders were adjusted for and why they were included | Results, Table 2, Figure 1 |
|  |  | (b) Report category boundaries when continuous variables were categorized | Methods |
|  |  | (c) If relevant, consider translating estimates of relative risk into absolute risk for a meaningful time period | Results, Table 2 |
| Other analyses | 17 | Report other analyses done—e.g. analyses of subgroups and interactions, and sensitivity analyses | Results, Appendix Table 6 |
| Discussion | | | |
| Key results | 18 | Summarize key results with reference to study objectives | Discussion |
| Limitations | 19 | Discuss limitations of the study, taking into account sources of potential bias or imprecision. Discuss both direction and magnitude of any potential bias | Discussion |
| Interpretation | 20 | Give a cautious overall interpretation of results considering objectives, limitations, multiplicity of analyses, results from similar studies, and other relevant evidence | Discussion |
| Generalizability | 21 | Discuss the generalizability (external validity) of the study results | Discussion |
| Other information | | | |
| Funding | 22 | Give the source of funding and the role of the funders for the present study and, if applicable, for the original study on which the present article is based | Funding/support |

**Additional file 1**: **Table S2. Coding definitions for comorbid conditions, outcomes and exposures**

| Variable | Database | Code/Definition |
| --- | --- | --- |
| Comorbidities | | |
| Dementia | CIHI-DAD | **ICD-9** 2900, 2901, 2903, 2904, 2908, 2909, 2948, 2949, 3310, 3311, 3312, 2941, 797 **ICD-10** F065, F066, F068, F069, F09, F00, F01, F02, F03, F051, G30, G31, R54 |
|  | OMHRS | **DSM-IV** 29040, 29041, 29042, 29043, 29120, 29282, 29410, 29411, 29480, 78090 |
|  | OHIP | 290, 331, 797 |
| Schizophrenia or other psychotic disorder | CIHI-DAD | **ICD-9** 2950, 2951, 2952, 2953, 2954, 2955, 2956, 2957, 2958, 2959, 2970, 2971, 2972, 2973, 2978, 2979, 2980, 2981, 2983, 2984, 2988, 2989  **ICD-10** F060, F062, F105, F107, F115, F117, F125, F127, F135, F137, F145, F147, F155, F157, F165, F167, F175, F177, F185, F187, F195, F197, F200, F201, F202, F203, F204, F205, F206, F208, F209, F220, F228, F229, F230, F231, F232, F233, F238, F239, F24, F250, F251, F252, F258, F259, F28, F29 |
|  | OMHRS | **DSM-IV** 29130, 29150, 29211, 29212, 29381, 29382, 29510, 29520, 29530, 29540, 29560, 29570, 29590, 29710, 29730, 29880, 29890 |
|  | OHIP | 291, 292, 295, 297, 298, Q021 |
| Bipolar disorder | CIHI-DAD | **ICD-9** 2960, 2961, 2964, 2965, 2966, 2967, 2968  **ICD-10** F300, F301, F302, F308, F309, F310, F311, F312, F313, F314, F315, F316, F317, F318, F319 |
|  | OMHRS | **DSM-IV** 29600, 29601, 29602, 29603, 29604, 29605, 29606, 29640, 29641, 29642, 29643, 29644, 29645, 29646, 29650, 29651, 29652, 29653, 29654, 29655, 29656, 29660, 29661, 29662, 29663, 29664, 29665, 29666, 29670, 29680, 29689 |
|  | OHIP | 296, Q020 |
| Major depression and/or anxiety disorder | CIHI-DAD | **ICD-9** 2962, 2963, 3000, 3002, 3003, 3004, 3091, 311  **ICD-10** F063, F064, F320, F321, F322, F323, F328, F329, F330, F331, F332, F333, F334, F338, F339, F341, F400, F401, F402, F408, F409, F410, F411, F412, F413, F418, F419, F420, F421, F422, F428, F429, F430, F431 |
|  | OMHRS | **DSM-IV** 29189, 29284, 29289, 29383, 29384, 29620, 29621, 29622, 29623, 29624, 29625, 29626, 29630, 29631, 29632, 29633, 29634, 29635, 29636, 30000, 30001, 30002, 30021, 30022, 30023, 30029, 30030, 30040, 30113 |
|  | OHIP | 311 |
| Parkinson’s disease | CIHI-DAD | **ICD-9** 332  **ICD-10** G20, F023 |
|  | OMHRS | 332 |
| Congestive heart failure | CIHI-DAD | **ICD-9** 425, 5184, 514, 428  **ICD-10** I500, I501, I509, I255, J81  **CCP** 4961, 4962, 4963, 4964  **CCI** 1HP53, 1HP55, 1HZ53GRFR, 1HZ53LAFR, 1HZ53SYFR |
|  | OHIP | 428, R701, R702, Z429 |
| Chronic kidney disease | CIHI-DAD | **ICD-9** 4030, 4031, 4039, 4040, 4041, 4049, 582, 583, 580, 581, 584, 585, 586, 587, 5880, 5888, 5889, 5937  **ICD-10** E102, E112, E132, E142, I12, I13, N08, N18, N19 |
|  | OHIP | 403, 585 |
| Hypertension | CIHI-DAD | **ICD-9** 401, 402, 403, 404, 405  **ICD10** I10, I11, I12, I13, I15 |
|  | OHIP | 401, 402, 403, |
| Chronic liver disease | CIHI-DAD | **ICD-9** 4561, 4562, 070, 5722, 5723, 5724, 5728, 573, 7824, V026, 2750, 2751, 7891, 7895, 571  **ICD-10** B16, B17, B18, B19, I85, R17, R18, R160, R162, B942, Z225, E831, E830, K70, K713, K714, K715, K717, K721, K729, K73, K74, K753, K754, K758, K759, K76, K77 |
|  | OHIP | 571, 573, 070, Z551, Z554 |
| Hypothyroidism | CIHI-DAD | **ICD-9** 243, 2440, 2441, 2442, 2443, 2448, 2449  **ICD-10** E030, E031, E032, E033, E034, E035, E038, E039, E890 |
|  | OHIP | 243, 244 |
| Cancer | CIHI-DAD | **ICD-9** 150, 154, 155, 157, 162, 174, 175, 185, 203, 204, 205, 206, 207, 208  **ICD-10** 971, 980, 982, 984, 985, 986, 987, 988, 989, 990, 991, 993, C15, C18, C19, C20, C22, C25, C34, C50, C56, C61, C82, C83, C85, C91, C92, C93, C94, C95, C00, D05 |
|  | OHIP | 203, 204, 205, 206, 207, 208, 150, 154, 155, 157, 162, 174, 175, 183, 185 |
| Diabetes Mellitus | CIHI-DAD | **ICD-9** 250  **ICD-10** E10, E11, E13, E14 |
|  | OHIP | 250, K029, K030, Q040 |
| Pneumonia | CIHI-DAD | **ICD-9** 480, 481, 482, 483, 484, 485, 486, 7700  **ICD-10** J12, J13, J14, J15, J16, J17, J18, P23 |
| Coronary artery disease | CIHI-DAD | **ICD-9** 410 412, 414, 4292, 4295, 4296, 4297  **ICD-10** I21, I22, I23, I24, I25, Z955, Z958, Z959, R931, T822  **CCP** 4801, 4802, 4803, 4804, 4805, 481, 482, 483  **CCI** 1IJ26, 1IJ27, 1IJ54, 1IJ57, 1IJ50, 1IJ76 |
|  | OHIP | 410, 412, R741, R742, R743, G298, E646, E651, E652, E654, E655, G262, Z434, Z448 |
| Angina | CIHI-DAD | **ICD-9** 413  **ICD-10** I20 |
|  | OHIP | 413 |
| Atrial fibrillation/flutter | CIHI-DAD | **ICD-9** 4273  **ICD-10** I48 |
| Previous hyponatremia | CIHI-DAD | **ICD-9** 2761  **ICD-10** E871 |
| Myocardial Infarction |  | **ICD-9** 410  **ICD-10** I21, I22 |
| Haemorrhagic stroke | CIHI-DAD | **ICD-9** 430, 431  **ICD-10** I600, I601, I602, I603, I604, I605, I606, I607, I609, I61 |
| Ischemic stroke | CIHI-DAD | **ICD-9** 436, 4340, 4341, 4349, 3623  **ICD-10** I630, I631, I632, I633, I634, I635, I638, I639, I64, H341 |
| Transient ischemic attack | CIHI-DAD | **ICD-9** 435  **ICD-10** G450, G451, G452, G453, G458, G459, H340 |
| Chronic lung disease | CIHI-DAD | **ICD-9** 491, 492, 493, 494, 495, 496, 500, 501, 502, 503, 504, 505, 5064, 5069, 5081, 515, 516, 517, 5185, 5188, 5198, 5199, 4168, 4169  **ICD-10** I272, I278, I279, J40, J41, J42, J43, J44, J45, J47, J60, J61, J62, J63, J64, J65, J66, J67, J68, J701, J703, J704, J708, J709, J82, J84, J92, J941, J949, J953, J961, J969, J984, J988, J989, J99 |
|  | OHIP | 491, 492, 493, 494, 496, 501, 502, 515, 518, 519  J689, J889 |
| Seizure | CIHI-DAD | **ICD-9** 345, 7803  **ICD-10** G40, G41, R560, R568 |
| Acute kidney injury | CIHI-DAD | **ICD-9** 584  **ICD-10** N17 |
| Hypotension | CIHI-DAD | **ICD-9** 458  **ICD-10** I95 |
| Acute urinary retention | CIHI-DAD | **ICD-9** 7882  **ICD-10** R33 |
| Delirium | CIHI-DAD | **ICD-9** 293  **ICD-10** F05 |
| Peripheral vascular disease | CIHI-DAD | **ICD-9** 4402, 4408, 4409, 5571, 4439, 444  **ICD-10** I700, I702, I708, I709, I731, I738, I739, K551  **CCP** 5125, 5129, 5014, 5016, 5018, 5028, 5038  **CCI** 1KA76, 1KA50, 1KE76, 1KG26, 1KG50, 1KG57, 1KG76MI, 1KG87 |
|  | OHIP | R787, R780, R797, R804, R809, R875, R815, R936, R783, R784,R785, E626, R814, R786, R937, R860, R861, R855, R856, R933, R934, R791, E672, R794, R813, R867, E649 |
| Outcomes | | |
| Hyponatremia^*^ | CIHI-DAD | **ICD-10** E871 |
| Bowel obstruction | CIHI-DAD | **ICD-10** K56 |
| Exposures |  |  |
| Risperidone | ODB | Higher dose: >0.5 mg/day; Normal dose ≤0.5 mg/day |
| Olanzapine |  | Higher dose: >2.5 mg/day; Normal dose ≤2.5 mg/day |
| Quetiapine |  | Higher dose: >25 mg/day; Normal dose ≤25 mg/day |
| CCI=Canadian Classification of Health Interventions. CCP=Canadian Classification of Diagnostic, Therapeutic and Surgical Procedures. CIHI-DAD=Canadian Institute for Health Information Discharge Abstract Database. ICD-9=International Classification of Diseases, Ninth Revision. ICD-10=International Classification of Diseases, Tenth Revision. Ontario Drug Benefit database=ODB. OHIP=Ontario Health Insurance Plan database. OMHRS=Ontario Mental Health Reporting System database. RPDB=Ontario’s Registered Persons Database. *Validation of the code for hyponatremia was performed on approximately 64 499 hospitalizations with linked laboratory measurements for serum sodium. See Methods section for a description of the validation.(30) | | |

**Additional file 1**: **Table S3. Variables included in propensity score model**

| Demographics | Age, sex, rural neighborhood |
| --- | --- |
| Income |  |
| Index date |  |
| Residential status (community-dwelling or long-term care) |  |
| Comorbid conditions | Charlson comorbidity index, Johns Hopkins ACG System Aggregated Diagnosis Groups, dementia, schizophrenia, bipolar disorder, unipolar depression/anxiety, Parkinson’s disease, congestive heart failure, chronic kidney disease, hypertension, chronic liver disease, hypothyroidism, cancer, diabetes mellitus, pneumonia, coronary artery disease, angina, atrial fibrillation/flutter, myocardial infarction, hemorrhagic stroke, ischemic stroke, transient ischemic attack, chronic lung disease, seizure, acute kidney injury, hypotension, acute urinary retention, peripheral vascular disease |
| Concurrent medication use | Number of unique drug products, anticonvulsant, antidepressant, antidiabetics, antineoplastic, thyroxin, potassium sparing diuretic, non-potassium sparing diuretic, ACE inhibitor and/or ARB, NSAID (excluding aspirin), calcium channel blocker, beta-adrenergic antagonist, statin, benzodiazepine, digoxin, overactive bladder medication, antibiotic, warfarin, anticoagulant, antiplatelet, acetylcholine inhaler, corticosteroid inhaler, beta-agonist inhaler, cholinesterase inhibitor, lithium, glucose tests strips. |
| Number of healthcare contacts | Hospitalization, emergency department visit, family physician visit, psychiatrist visit, geriatrician visit, neurologist visit, nephrologist visit, cardiologist visit, urologist visit, obstetrician/gynecologist visit |
| Number of healthcare uses | Previous sodium tests, carotid ultrasound, cardiac catheterization, echocardiography, holter monitoring, cardiac stress test, coronary endarterectomy, colorectal cancer screening, cervical cancer screening, prostate-specific antigen test, mammography, flu shot, bone mineral density test, hearing test, cystoscopy, cataract surgery, computed tomography of the head, computed tomography of the neck, computed tomography of the thorax, computed tomography of the abdomen, computed tomography of the pelvis, computed tomography of the spine, computed tomography of the extremities, chest x-ray, pulmonary function test, electroencephalography, urine culture, heart valve replacement, at-home physician service, cholesterol test |
| ACE inhibitor=angiotensin-converting enzyme inhibitor. ACG=adjusted clinical groups. ARB=angiotensin II receptor blocker. NSAID=non-steroidal anti-inflammatory drug. | |

Remaining

*26 619* Discharged from a hospital in the two days prior to the index date

*1081* Evidence of end-stage renal disease prior to the index date

**Matched (1:1)**

***58 008*** antipsychotic users

***58 008*** antipsychotic non-users

*608, 147* Ontario residents >65 years of age with evidence of a baseline psychiatric disorder who were not dispensed a new oral outpatient prescription for any antipsychotic medication in the six months prior to the randomly assigned index date (from June 2003 to March 2012)

*119, 790* Ontario residents >65 years of age with evidence of a baseline psychiatric disorder who were dispensed a new oral outpatient prescription for one of the following study atypical antipsychotic medications prior to the index date: quetiapine, olanzapine, risperidone (from June 2003 to March 2012)

*409 945* Without at least one outpatient medication dispensed in the 90 days prior to the index date

*16 325* Discharged from a hospital in the two days prior to the index date

*6041* Evidence of end-stage renal disease prior to the index date

Excluded

***92 090***

***175 836***

**Additional file 1**: **Figure S1. Cohort selection**

##### Additional file 1: Table S4. Full baseline characteristics of atypical antipsychotic medication users and non-users^a^

| Characteristic | Unmatched | | | Matched | | |
| --- | --- | --- | --- | --- | --- | --- |
|  | **Antipsychotic**  **users (n=92 090)** | **Antipsychotic**  **non-users**  **(n=175 836)** | **Standardized Difference,%^b^** | **Antipsychotic**  **users (n=58,008)** | **Antipsychotic**  **non-users**  **(n=58,008)** | **Standardized Difference,%^b^** |
| Demographic |  |  |  |  |  |  |
| Age, mean (SD), years | 81 (7.80) | 79 (7.95) | 29.21% | 81 (7.71) | 81 (7.71) | 0.26% |
| Women | 58 647 (63.68) | 111 968 (63.68) | 0.01% | 38 736 (66.78) | 38 736 (66.78) | 0.00% |
| Income quintile^c^ |  |  |  |  |  |  |
| 1 (low) | 20 160 (21.89) | 37 436 (21.29) | 1.46% | 12 331 (21.26) | 13 081 (22.55) | 3.13% |
| 2 | 18 854 (20.47) | 36 395 (20.70) | 0.56% | 11 888 (20.49) | 12 057 (20.79) | 0.72% |
| 3 (medium) | 17 999 (19.55) | 33 861 (19.26) | 0.73% | 11 630 (20.05) | 11 408 (19.67) | 0.96% |
| 4 | 17 607 (19.12) | 33 206 (18.88) | 0.60% | 11 213 (19.33) | 10 847 (18.70) | 1.61% |
| 5 (high) | 17 058 (18.52) | 34 373 (19.55) | 2.61% | 10 946 (18.87) | 10 615 (18.30) | 1.47% |
| Year of cohort entry |  |  |  |  |  |  |
| 2003-2004 | 19 653 (21.34) | 31 335 (17.82) | 8.88% | 11 417 (19.68) | 11 312 (19.50) | 0.46% |
| 2005-2006 | 21 756 (23.62) | 38 010 (21.62) | 4.80% | 13 395 (23.09) | 13 430 (23.15) | 0.14% |
| 2007-2008 | 18 970 (20.60) | 35 337 (20.09) | 1.27% | 11 962 (20.62) | 11 762 (20.28) | 0.85% |
| 2009-2010 | 19 814 (21.52) | 42 371 (24.10) | 6.15% | 13 317 (22.96) | 13 341 (23.00) | 0.10% |
| 2011-2012 | 11 897 (12.92) | 28 783 (16.37) | 9.77% | 7 917 (13.65) | 8 163 (14.07) | 1.23% |
| Rural residence | 11 759 (12.77) | 23 484 (13.36) | 1.74% | 7 671 (13.22) | 7 557 (13.03) | 0.58% |
| Long-term care | 32 644 (35.45) | 26 705 (15.19) | 47.91% | 16 409 (28.29) | 16 409 (28.29) | 0.00% |
| Prescribing Physician |  |  |  |  |  |  |
| Family Physician | 64 900 (70.47) |  |  | 40 829 (70.39) |  |  |
| Psychiatrist | 7420 (8.06) |  |  | 3989 (6.88) |  |  |
| Geriatrician | 4387 (4.76) |  |  | 3356 (5.79) |  |  |
| Neurologist | 1943 (2.11) |  |  | 1367 (2.36) |  |  |
| Other | 3383 (3.67) |  |  | 2139 (3.69) |  |  |
| Missing | 10 057 (10.92) |  |  | 6328 (10.91) |  |  |
| Comorbid conditions^d^ |  |  |  |  |  |  |
| Charlson comorbidity index, mean (SD) | 1.68 (1.79) | 1.56 (1.78) | 6.72% | 0.87 (1.47) | 0.94 (1.53) | 4.67% |
| Johns Hopkins ACG System Aggregated Diagnosis Groups, mean (SD) | 13.90 (4.17) | 13.69 (3.96) | 5.17% | 13.37 (4.16) | 13.69 (4.07) | 7.78% |
| Dementia | 71 933 (78.11) | 92 049 (52.35) | 56.19% | 44 715 (77.08) | 44 715 (77.08) | 0.00% |
| Schizophrenia | 14 838 (16.11) | 14 072 (8.00) | 25.10% | 4 756 (8.20) | 4 756 (8.20) | 0.00% |
| Bipolar disorder | 10 174 (11.05) | 11 377 (6.47) | 16.25% | 3 295 (5.68) | 3 295 (5.68) | 0.00% |
| Unipolar depression/anxiety | 28 419 (30.86) | 74 574 (42.41) | 24.15% | 15 038 (25.92) | 15 038 (25.92) | 0.00% |
| Parkinson’s disease | 8652 (9.40) | 19 015 (10.81) | 4.71% | 3 780 (6.52) | 3 780 (6.52) | 0.00% |
| Congestive heart failure | 19 029 (20.66) | 33 627 (19.12) | 3.86% | 10 038 (17.30) | 10 038 (17.30) | 0.00% |
| Chronic kidney disease | 8127 (8.83) | 15 323 (8.71) | 0.39% | 3 140 (5.41) | 3 140 (5.41) | 0.00% |
| Hypertension | 65 205 (70.81) | 131 562 (74.82) | 9.03% | 40 929 (70.56) | 40 419 (69.68) | 1.92% |
| Chronic liver disease | 2980 (3.24) | 6388 (3.63) | 2.18% | 1 664 (2.87) | 1 807 (3.12) | 1.45% |
| Hypothyroidism | 10 213 (11.09) | 20 354 (11.58) | 1.53% | 6 222 (10.73) | 6 198 (10.68) | 0.13% |
| Cancer | 12 145 (13.19) | 25 758 (14.65) | 4.22% | 7 321 (12.62) | 7 864 (13.56) | 2.78% |
| Diabetes mellitus | 14 245 (15.47) | 30 491 (17.34) | 5.06% | 17 590 (30.32) | 18 457 (31.82) | 3.23% |
| Pneumonia | 8006 (8.69) | 12 843 (7.30) | 5.12% | 4 237 (7.30) | 4 755 (8.20) | 3.34% |
| Coronary artery disease^e^ | 31 417 (34.12) | 61 334 (34.88) | 1.61% | 18 641 (32.14) | 19 184 (33.07) | 2.00% |
| Angina | 20 496 (22.26) | 42 264 (24.04) | 4.22% | 12 166 (20.97) | 12 462 (21.48) | 1.25% |
| Atrial fibrillation/flutter | 9841 (10.69) | 17 100 (9.72) | 3.18% | 5 428 (9.36) | 5 613 (9.68) | 1.09% |
| Previous hyponatremia | 3403 (3.70) | 5416 (3.08) | 3.40% | 1 766 (3.04) | 2 111 (3.64) | 3.31% |
| Myocardial infarction | 4741 (5.15) | 9010 (5.12) | 0.11% | 2 585 (4.46) | 2 696 (4.65) | 0.92% |
| Hemorrhagic stroke | 698 (0.76) | 935 (0.53) | 2.83% | 331 (0.57) | 404 (0.70) | 1.59% |
| Ischemic stroke | 5328 (5.79) | 7628 (4.34) | 6.61% | 2725 (4.70) | 3 340 (5.76) | 4.76% |
| Transient ischemic attack | 1818 (1.97) | 3074 (1.75) | 1.67% | 1 023 (1.76) | 1 176 (2.03) | 1.93% |
| Lung disease | 26 237 (28.49) | 53 842 (30.62) | 4.67% | 15 489 (26.70) | 16 891 (29.12) | 5.39% |
| Seizure | 1782 (1.94) | 2780 (1.58) | 2.69% | 879 (1.52) | 1 087 (1.87) | 2.78% |
| Acute kidney injury | 3453 (3.75) | 5616 (3.19) | 3.04% | 1 482 (2.55) | 1 501 (2.59) | 0.21% |
| Hypotension | 2704 (2.94) | 4572 (2.60) | 2.05% | 1 320 (2.28) | 1 398 (2.41) | 0.89% |
| Acute urinary retention | 3337 (3.62) | 5179 (2.95) | 3.81% | 1650 (2.84) | 1 774 (3.06) | 1.26% |
| Delirium | 7112 (7.72) | 6013 (3.42) | 18.84% | 3424 (5.90) | 2546 (4.39) | 6.85% |
| Peripheral vascular disease | 1939 (2.11) | 4061 (2.31) | 1.39% | 1043 (1.80) | 1238 (2.13) | 2.42% |
| Concurrent medication use^f^ |  |  |  |  |  |  |
| Number of unique drug products, mean (SD) | 9.71 (6.39) | 8.94 (5.46) | 13.00% | 8.91 (5.92) | 9.41 (5.61) | 8.67% |
| Anticonvulsants | 10 970 (11.91) | 16 409 (9.33) | 8.38% | 5552 (9.57) | 6688 (11.53) | 6.38% |
| Antidepressants | 46 600 (50.60) | 65 227 (37.10) | 27.48% | 25 197 (43.44) | 26 871 (46.32) | 5.80% |
| Antidiabetics | 14 245 (15.47) | 21 969 (12.49) | 8.59% | 8526 (14.70) | 9307 (16.04) | 3.72% |
| Antineoplastics | 3240 (3.52) | 6863 (3.90) | 2.04% | 1958 (3.38) | 2151 (3.71) | 1.80% |
| Thyroxine | 16 580 (18.00) | 33 499 (19.05) | 2.70% | 10 406 (17.94) | 10 846 (18.70) | 1.96% |
| Potassium sparing diuretics | 5390 (5.85) | 11 025 (6.27) | 1.75% | 3267 (5.63) | 3236 (5.58) | 0.23% |
| Non-potassium sparing diuretics | 31 051 (33.72) | 61 131 (34.77) | 2.21% | 18 611 (32.08) | 18 665 (32.18) | 0.20% |
| ACE inhibitors and/or ARBs | 39 596 (43.00) | 88 563 (50.37) | 14.81% | 24 853 (42.84) | 25 660 (44.24) | 2.81% |
| NSAIDs (excl. ASA) | 13 638 (14.81) | 30 146 (17.14) | 6.38% | 8667 (14.94) | 9047 (15.60) | 1.82% |
| Calcium channel blockers | 22 902 (24.87) | 49 786 (28.31) | 7.80% | 14 642 (25.24) | 15 011 (25.88) | 1.46% |
| Beta-adrenergic agonists | 26 099 (28.34) | 55 259 (31.43) | 6.74% | 16 169 (27.87) | 16 254 (28.02) | 0.33% |
| Statins | 29 558 (32.10) | 72 878 (41.45) | 19.48% | 19 171 (33.05) | 19 514 (33.64) | 1.25% |
| Benzodiazepines | 33 031 (35.87) | 45 988 (26.15) | 21.12% | 17 616 (30.37) | 18 692 (32.22) | 4.00% |
| Digoxin | 5923 (6.43) | 10 434 (5.93) | 2.07% | 3 523 (6.07) | 3848 (6.63) | 2.30% |
| Overactive bladder | 5188 (5.63) | 8652 (4.92) | 3.19% | 3 094 (5.33) | 3311 (5.71) | 1.64% |
| Antibiotics | 35 094 (38.11) | 63 965 (36.38) | 3.58% | 20 856 (35.95) | 21 900 (37.75) | 3.73% |
| Warfarin | 9610 (10.44) | 19 752 (11.23) | 2.57% | 5 761 (9.93) | 6189 (10.67) | 2.43% |
| Anticoagulents | 895 (0.97) | 1287 (0.73) | 2.61% | 476 (0.82) | 463 (0.80) | 0.25% |
| Antiplatlets | 6665 (7.24) | 13 455 (7.65) | 1.58% | 4009 (6.91) | 4511 (7.78) | 3.32% |
| Acetylcholine inhalers | 6454 (7.01) | 13 153 (7.48) | 1.82% | 3731 (6.43) | 4319 (7.45) | 3.99% |
| Corticosteroid inhalers | 5309 (34.21) | 11 301 (13.84) | 49.09% | 3183 (5.49) | 3647 (6.29) | 3.40% |
| Beta-agonist inhalers | 11 801 (12.81) | 25 019 (14.23) | 4.14% | 6974 (12.02) | 8061 (13.90) | 5.58% |
| Cholinesterase inhibitors | 31 505 (34.21) | 24 342 (13.84) | 49.09% | 18 139 (31.27) | 16 804 (28.97) | 5.02% |
| Lithium | 1176 (1.28) | 1042 (0.59) | 7.12% | 367 (0.63) | 412 (0.71) | 0.95% |
| Glucose test strips | 10 411 (11.31) | 23 666 (13.46) | 6.54% | 6162 (10.62) | 6784 (11.69) | 3.41% |
| Healthcare contacts, mean (SD)^g^ |  |  |  |  |  |  |
| Hospitalizations | 0.51 (0.92) | 0.32 (0.76) | 22.62% | 0.40 (0.78) | 0.39 (0.82) | 1.25% |
| Emergency department visits | 1.29 (2.05) | 0.84 (1.60) | 24.66% | 1.04 (1.57) | 1.00 (1.70) | 2.45% |
| Family physician visits | 18.57 (17.99) | 13.61 (13.10) | 31.91% | 15.84 (15.53) | 16.30 (15.33) | 2.98% |
| Geriatrician visits | 0.82 (3.56) | 0.34 (2.12) | 16.90% | 0.58 (2.48) | 0.49 (2.43) | 3.67% |
| Psychiatrist visits | 1.69 (7.47) | 0.36 (2.52) | 26.63% | 0.64 (2.41) | 0.40 (2.16) | 10.0% |
| Neurologist visits | 0.43 (2.18) | 0.29 (1.23) | 8.21% | 0.30 (1.18) | 0.31 (1.34) | 0.79% |
| Nephrologist visits | 0.16 (1.08) | 0.15 (1.04) | 0.94% | 0.11 (0.86) | 0.12 (0.94) | 1.11% |
| Cardiologist visits | 1.23 (3.17) | 1.16 (2.93) | 2.30% | 1.03 (2.69) | 1.03 (2.72) | 0.00% |
| Urologist visits | 0.32 (1.46) | 0.33 (1.34) | 0.71% | 0.28 (1.33) | 0.29 (1.27) | 0.77% |
| Obstetrician/Gynecologist visits | 0.07 (0.58) | 0.09 (0.63) | 3.31% | 0.07 (0.53) | 0.07 (0.53) | 0.00% |
| Healthcare use^h^ |  |  |  |  |  |  |
| Previous sodium tests | 63 335 (68.78) | 79 930 (45.46) | 48.48% | 38 190 (64.84) | 38 145 (65.76) | 0.16% |
| Carotid ultrasound | 4568 (4.96) | 9137 (5.20) | 1.07% | 2 721 (4.69) | 2 883 (4.97) | 1.30% |
| Cardiac catheterization | 742 (0.81) | 2334 (1.33) | 5.08% | 414 (0.71) | 477 (0.82) | 1.24% |
| Echocardiography | 12 411 (13.48) | 27 264 (15.51) | 5.76% | 7 247 (12.49) | 7 355 (12.68) | 0.56% |
| Holter monitoring | 4818 (5.23) | 10 904 (6.20) | 4.18% | 2 962 (5.11) | 3 032 (5.23) | 0.55% |
| Cardiac stress test | 5757 (6.25) | 15 993 (9.10) | 10.70% | 3 542 (6.11) | 3 700 (6.38) | 1.13% |
| Coronary endarterectomy | 45 (0.05) | 112 (0.06) | 0.63% | 32 (0.06) | 31 (0.05) | 0.07% |
| Colorectal cancer screening | 10 653 (11.57) | 29 013 (16.50) | 14.24% | 6 767 (11.67) | 6 823 (11.76) | 0.30% |
| Cervical cancer screening | 2047 (2.22) | 8290 (4.71) | 13.65% | 1 447 (2.49) | 1 427 (2.46) | 0.22% |
| Prostate specific antigen test | 1147 (1.25) | 4580 (2.60) | 9.90% | 770 (1.33) | 792 (1.37) | 0.33% |
| Mammography | 3507 (3.81) | 12 320 (7.01) | 14.18% | 2 425 (4.18) | 2 370 (4.09) | 0.48% |
| Flu shot | 42 439 (46.08) | 95 645 (54.39) | 16.68% | 27 859 (48.03) | 27 553 (47.50) | 1.06% |
| Thyroid stimulating hormone | 57 414 (62.35) | 100 900 (57.38) | 10.14% | 34 911 (60.18) | 34 766 (59.93) | 0.51% |
| Bone mineral density test | 5792 (6.29) | 18 558 (10.55) | 15.40% | 4 040 (6.96) | 4 110 (7.09) | 0.47% |
| Hearing test | 3705 (4.02) | 9327 (5.30) | 6.08% | 2 375 (4.09) | 2 537 (4.37) | 1.39% |
| Cytoscopy | 3959 (4.30) | 7739 (4.40) | 0.50% | 2 253 (3.88) | 2 325 (4.01) | 0.64% |
| Cataract surgery | 3233 (3.51) | 8372 (4.76) | 6.28% | 2 137 (3.68) | 2 122 (3.66) | 0.14% |
| Computed tomography of the head | 26 927 (29.24) | 25 724 (14.63) | 35.87% | 13 261 (22.86) | 12 896 (22.23) | 1.51% |
| Computed tomography of the neck | 741 (0.80) | 1309 (0.74) | 0.69% | 417 (0.72) | 469 (0.81) | 1.03% |
| Computed tomography of the thorax | 5054 (5.49) | 9369 (5.33) | 0.71% | 2 638 (4.55) | 3 175 (5.47) | 4.24% |
| Computed tomography of the abdomen | 7773 (8.44) | 13 668 (7.77) | 2.45% | 4 109 (7.08) | 4 574 (7.89) | 3.05% |
| Computed tomography of the pelvis | 7177 (7.79) | 12 371 (7.04) | 2.89% | 3 799 (6.55) | 4 165 (7.18) | 2.50% |
| Computed tomography of the spine | 2003 (2.18) | 3474 (1.98) | 1.40% | 1 030 (1.78) | 1 106 (1.91) | 0.97% |
| Computed tomography of the extremities | 675 (0.73) | 1085 (0.62) | 1.42% | 368 (0.63) | 392 (0.68) | 0.51% |
| Chest x-ray | 43 501 (47.24) | 69 077 (39.28) | 16.10% | 24 600 (42.41) | 25 086 (43.25) | 1.69% |
| Pulmonary function test | 4882 (5.30) | 13 764 (7.83) | 10.21% | 2 901 (5.00) | 3 404 (5.87) | 3.83% |
| Electroencephalography | 1664 (1.81) | 1836 (1.04) | 6.44% | 791 (1.36) | 826 (1.42) | 0.51% |
| Urine culture | 35 750 (38.82) | 49 370 (28.08) | 22.92% | 20 385 (35.14) | 19 477 (33.58) | 3.30% |
| Heart valve replacement | 72 (0.08) | 208 (0.12) | 1.28% | 43 (0.07) | 32 (0.06) | 0.75% |
| At-home physician services | 11 389 (12.37) | 11 474 (6.53) | 20.07% | 5 832 (10.05) | 5 684 (9.80) | 0.85% |
| Cholesterol tests | 33 009 (35.84) | 83 296 (47.37) | 23.55% | 21 686 (37.38) | 20 975 (36.16) | 2.54% |
| Laboratory measurements^i^ |  |  |  |  |  |  |
| Most recent serum sodium, N (%) | 14 346 (15.58) | 21 948 (23.83) | 20.87% | 7 242 (12.48) | 7 242 (12.48) | 0.00% |
| Most recent serum sodium, mean (SD) | 140.32 (3.47) | 140.41 (3.22) | 2.69% | 140.43 (3.42) | 140.28 (3.41) | 4.39% |

ACE inhibitor=angiotensin-converting enzyme inhibitor. ACG=adjusted clinical groups. ARB=angiotensin II receptor blocker. IQR=interquartile range. NSAID=Non-steroidal anti-inflammatory drug. SD=standard deviation.

a Data are presented as the number (percentage) of patients, unless otherwise reported.

b Standardized differences are less sensitive to sample size than traditional hypothesis tests. They provide a measure of the difference between groups with respect to the pooled standard deviation; a standardized difference greater than 10% was considered as a meaningful difference between the groups.
c Income was categorized into fifths of average neighborhood income on the index date.

d Comorbid conditions in the five years preceding the index date were considered.

e Coronary artery disease includes receipt of coronary artery bypass graft surgery, and percutaneous coronary intervention.

f Concurrent medication use in the six months preceding the index date were considered.

g Healthcare contacts in the year preceding the index date were considered.

h Healthcare use in the year preceding the index date was considered.

i Serum sodium measurements were obtained at a mean (SD) of 140 (102) days in users and 149 (101) days in non-users, prior to the index date.

**Additional file 1**: **Table S5. Baseline characteristics of matched atypical antipsychotic users and non-users at 90 days prior to the index date^a^**

| Characteristic | Matched | | |
| --- | --- | --- | --- |
|  | **Antipsychotic**  **users**  **(n=42 698)** | **Antipsychotic**  **non-users**  **(n=42 698)** | **Standardized Difference,%^b^** |
| Demographic |  |  |  |
| Age, mean (SD), years | 81 (7.56) | 81 (7.57) | 0.26% |
| Women | 28 842 (67.55) | 28 842 (67.55) | 0.00% |
| Income quintile^c^ |  |  |  |
| 1 (low) | 9552 (22.37) | 8927 (20.91) | 3.56% |
| 2 | 8899 (20.84) | 8726 (20.44) | 1.00% |
| 3 (medium) | 8389 (19.65) | 8615 (20.18) | 1.33% |
| 4 | 8003 (18.74) | 8362 (19.58) | 2.14% |
| 5 (high) | 7855 (18.40) | 8068 (18.90) | 1.28% |
| Rural residence | 5698 (13.34) | 5586 (13.08) | 0.77% |
| Long-term care | 9303 (21.79) | 12 168 (28.50) | 15.51% |
| Comorbid conditions^d^ |  |  |  |
| Charlson comorbidity index, mean (SD) | 1.39 (1.58) | 1.49 (1.66) | 6.17% |
| Johns Hopkins ACG System Aggregated Diagnosis Groups, mean (SD) | 13.28 (4.09) | 13.61 (4.08) | 8.08% |
| Congestive heart failure | 6911 (16.19) | 7118 (16.67) | 1.31% |
| Chronic kidney disease | 2001 (4.69) | 2097 (4.91) | 1.05% |
| Hypertension | 30 047 (70.37) | 29 571 (69.26) | 2.43% |
| Chronic liver disease | 1159 (2.71) | 1234 (2.89) | 1.06% |
| Hypothyroidism | 4666 (10.93) | 4616 (10.81) | 0.38% |
| Cancer | 5154 (12.07) | 5612 (13.14) | 3.23% |
| Diabetes mellitus | 12 666 (29.66) | 13 389 (31.36) | 3.68% |
| Pneumonia | 2771 (6.49) | 3270 (7.66) | 4.56% |
| Coronary artery disease^e^ | 13 535 (31.70) | 13 873 (32.49) | 1.70% |
| Angina | 8930 (20.91) | 9049 (21.19) | 0.68% |
| Atrial fibrillation/flutter | 3600 (8.43) | 3925 (9.19) | 2.69% |
| Previous hyponatremia | 1129 (2.64) | 1493 (3.50) | 4.94% |
| Myocardial infarction | 1703 (3.99) | 1835 (4.30) | 1.55% |
| Hemorrhagic stroke | 222 (0.52) | 290 (0.68) | 2.06% |
| Ischemic stroke | 1771 (4.15) | 2411 (5.65) | 6.95% |
| Transient ischemic attack | 745 (1.74) | 827 (1.94) | 1.43% |
| Lung disease | 11 061 (25.91) | 12 066 (28.26) | 5.30% |
| Seizure | 616 (1.44) | 757 (1.77) | 2.63% |
| Acute kidney injury | 904 (2.12) | 963 (2.26) | 0.94% |
| Hypotension | 889 (2.08) | 979 (2.29) | 1.44% |
| Acute urinary retention | 1055 (2.47) | 1268 (2.97) | 3.07% |
| Delirium | 1815 (4.25) | 1993 (4.67) | 2.02% |
| Peripheral vascular disease | 682 (1.60) | 840 (1.97) | 2.80% |
| Concurrent medication use^f^ |  |  |  |
| Number of unique drug products, mean (SD) | 8.49 (5.57) | 9.15 (5.50) | 11.92% |
| Anticonvulsants | 3739 (8.76) | 4745 (11.11) | 7.88% |
| Antidepressants | 17 715 (41.49) | 19 285 (45.17) | 7.43% |
| Antidiabetics | 1389 (3.25) | 1734 (4.06) | 4.31% |
| Antineoplastics | 1364 (3.19) | 1518 (3.56) | 2.00% |
| Thyroxine | 7811 (18.29) | 7933 (18.58) | 0.74% |
| Potassium sparing diuretics | 2298 (5.38) | 2279 (5.34) | 0.20% |
| Non-potassium sparing diuretics | 13 157 (30.81) | 13 513 (31.65) | 1.80% |
| ACE inhibitors and/or ARBs | 18 563 (43.48) | 18 720 (43.84) | 0.74% |
| NSAIDs (excl. ASA) | 6379 (14.94) | 6737 (15.78) | 2.33% |
| Calcium channel blockers | 10 735 (25.14) | 10 881 (25.48) | 0.79% |
| Beta-adrenergic agonists | 11 686 (27.37) | 11 732 (27.48) | 0.24% |
| Statins | 14 073 (32.96) | 13 956 (32.69) | 0.58% |
| Benzodiazepines | 11 309 (26.49) | 13 273 (31.09) | 10.17% |
| Overactive bladder | 2308 (5.41) | 2406 (5.63) | 1.01% |
| Antibiotics | 14 310 (33.51) | 15 890 (37.21) | 7.75% |
| Warfarin | 4407 (10.32) | 4050 (9.49) | 2.80% |
| Anticoagulents | 241 (0.56) | 327 (0.77) | 2.48% |
| Antiplatlets | 2874 (6.73) | 3132 (7.34) | 2.36% |
| Acetylcholine inhalers | 2515 (5.89) | 2944 (6.89) | 4.11% |
| Corticosteroid inhalers | 2257 (5.29) | 2687 (6.29) | 4.31% |
| Beta-agonist inhalers | 5694 (13.34) | 4801 (11.24) | 6.37% |
| Cholinesterase inhibitors | 12 415 (29.08) | 14 273 (33.43) | 9.40% |
| Lithium | 262 (0.61) | 273 (0.64) | 0.33% |
| Glucose test strips | 4239 (9.93) | 4866 (11.40) | 4.76% |
| Healthcare contacts, mean (SD)^g^ |  |  |  |
| Hospitalizations | 0.29 (0.68) | 0.36 (0.77) | 9.66% |
| Emergency department visits | 0.84 (1.42) | 0.93 (1.63) | 5.90% |
| Family physician visits | 14.22 (14.12) | 16.05 (15.17) | 12.50% |
| Geriatrician visits | 0.44 (2.04) | 0.48 (2.47) | 1.77% |
| Psychiatrist visits | 0.39 (1.84) | 0.38 (2.19) | 0.50% |
| Neurologist visits | 0.28 (1.02) | 0.31 (1.30) | 2.59% |
| Nephrologist visits | 0.08 (0.70) | 0.10 (0.78) | 2.70% |
| Cardiologist visits | 0.86 (2.30) | 0.96 (2.55) | 4.12% |
| Urologist visits | 0.25 (1.14) | 0.28 (1.18) | 2.59% |
| Obstetrician/Gynecologist visits | 0.07 (0.52) | 0.07 (0.51) | 0.00% |
| Healthcare use^h^ |  |  |  |
| Previous sodium tests | 27 877 (65.29) | 27 206 (63.72) | 3.28% |
| Carotid ultrasound | 1798 (4.21) | 2085 (4.88) | 3.23% |
| Cardiac catheterization | 278 (0.65) | 304 (0.71) | 0.74% |
| Echocardiography | 4654 (10.90) | 5124 (12.00) | 3.46% |
| Holter monitoring | 2055 (4.81) | 2189 (5.13) | 1.44% |
| Cardiac stress test | 2399 (5.62) | 2668 (6.25) | 2.67% |
| Coronary endarterectomy | 17 (0.04) | 15 (0.04) | 0.24% |
| Colorectal cancer screening | 4910 (11.50) | 5094 (11.93) | 1.34% |
| Cervical cancer screening | 1083 (2.54) | 1055 (2.47) | 0.42% |
| Prostate specific antigen test | 550 (1.29) | 560 (1.31) | 0.21% |
| Mammography | 1839 (4.31) | 1823 (4.27) | 0.18% |
| Flu shot | 21 271 (49.82) | 20 476 (47.96) | 3.73% |
| Thyroid stimulating hormone | 24 803 (58.09) | 25 398 (59.48) | 2.83% |
| Bone mineral density test | 3055 (7.15) | 3142 (7.36) | 0.79% |
| Hearing test | 1769 (4.14) | 1885 (4.41) | 1.34% |
| Cytoscopy | 1550 (3.63) | 1700 (3.98) | 1.84% |
| Cataract surgery | 1669 (3.91) | 1623 (3.80) | 0.56% |
| Computed tomography of the head | 7717 (18.07) | 8868 (20.77) | 6.82% |
| Computed tomography of the neck | 222 (0.52) | 296 (0.69) | 2.23% |
| Computed tomography of the thorax | 2017 (4.72) | 1528 (3.58) | 5.74% |
| Computed tomography of the abdomen | 2461 (5.76) | 3082 (7.22) | 5.91% |
| Computed tomography of the pelvis | 2256 (5.28) | 2802 (6.56) | 5.42% |
| Computed tomography of the spine | 632 (1.48) | 767 (1.80) | 2.49% |
| Computed tomography of the extremities | 204 (0.48) | 246 (0.58) | 1.36% |
| Chest x-ray | 15 965 (37.39) | 17 569 (41.15) | 7.70% |
| Pulmonary function test | 2014 (4.72) | 2385 (5.59) | 3.93% |
| Electroencephalography | 505 (1.18) | 575 (1.35) | 1.47% |
| Urine culture | 13 942 (32.65) | 14 178 (33.21) | 1.18% |
| Heart valve replacement | 14 (0.03) | 19 (0.04) | 0.60% |
| At-home physician services | 3706 (8.68) | 4035 (9.45) | 2.68% |
| Cholesterol tests | 15 636 (36.62) | 15 309 (35.85) | 1.59% |
| Laboratory measurements^i^ |  |  |  |
| Most recent serum sodium, N (%) | 5452 (12.77) | 5452 (12.77) | 0.00% |
| Most recent serum sodium, mean (SD) | 140.58 (3.30) | 140.35 (3.41) | 6.86% |

ACE inhibitor=angiotensin-converting enzyme inhibitor. ACG=adjusted clinical groups. ARB=angiotensin II receptor blocker. IQR=interquartile range. NSAID=Non-steroidal anti-inflammatory drug. SD=standard deviation.

a Data are presented as the number (percentage) of patients, unless otherwise reported.

b Standardized differences are less sensitive to sample size than traditional hypothesis tests. They provide a measure of the difference between groups with respect to the pooled standard deviation; a standardized difference greater than 10% was considered as a meaningful difference between the groups.
c Income was categorized into fifths of average neighborhood income on the index date.

d Comorbid conditions in the five years preceding the index date were considered.

e Coronary artery disease includes receipt of coronary artery bypass graft surgery, and percutaneous coronary intervention.

f Concurrent medication use in the six months preceding the index date were considered.

g Healthcare contacts in the year preceding the index date were considered.

h Healthcare use in the year preceding the index date was considered.

i Serum sodium measurements were obtained at a mean (SD) of 151 (104) days in users and 151 (105) days in non-users, prior to the index date.

**Additional file 1**: **Table S6. Risk factors for hospitalization with hyponatremia in antipsychotic medication users and non-users^*^**

|  | Antipsychotic users  (n=58 008) | Antipsychotic non-users  (n=58 008) |
| --- | --- | --- |
| Hospitalization with hyponatremia | **Relative risk (95% CI)** | **Relative risk (95% CI)** |
| Older age (per year) | 1.04 (1.01 to 1.07) | 1.02 (0.98 to 1.06) |
| Women (vs. men) | 1.36 (0.80 to 2.79) | 2.36 (1.09 to 5.12) |
| Chronic kidney disease (yes vs. no) | 1.44 (0.70 to 2.96) | 1.04 (0.37 to 2.97) |
| Congestive heart failure (yes vs. no) | 1.22 (0.73 to 2.04) | 1.13 (0.58 to 2.18) |
| Diabetes (yes vs. no) | 0.74 (0.45 to 1.21) | 1.09 (0.61 to 1.94) |
| Liver disease (yes vs. no) | 1.51 (0.54 to 4.20) | 1.18 (0.28 to 4.92) |
| Cancer (yes vs. no) | 1.84 (1.05 to 3.23) | 0.78 (0.32 to 1.94) |
| Hypothyroidism (yes vs. no) | 1.19 (0.65 to 2.16) | 0.71 (0.28 to 1.78) |
| Previous hyponatremia (yes vs. no) | 8.21 (4.93 to 13.66) | 7.99 (4.26 to14.95) |
| Diuretic use (yes vs. no) | 1.56 (0.98 to 2.50) | 1.85 (1.03 to 3.32) |
| Antiepileptic use (yes vs. no) | 1.25 (0.64 to 2.46) | 1.17 (0.52 to 2.63) |
| Antidepressant use (yes vs. no) | 1.24 (0.81 to 1.91) | 0.97 (0.56 to 1.68) |
| Antineoplastic use (yes vs. no) | 0.78 (0.24 to 2.58) | 1.83 (0.53 to 6.28) |

CI=confidence interval
* Separate multivariable logistic regression models created for antipsychotic users and non-users.
